# Anesthesia Assistance in Colonoscopy: Impact on Quality Indicators

**DOI:** 10.3389/fmed.2022.872231

**Published:** 2022-07-12

**Authors:** Min Liang, Xinyan Zhang, Chunhong Xu, Junli Cao, Zongwang Zhang

**Affiliations:** ^1^Jiangsu Province Key Laboratory of Anesthesiology, Xuzhou Medical University, Xuzhou, China; ^2^Jiangsu Province Key Laboratory of Anesthesia and Analgesia Application Technology, Xuzhou Medical University, Xuzhou, China; ^3^Department of Anesthesiology, Liaocheng People's Hospital, Liaocheng, China; ^4^Department of Pathology, Liaocheng People's Hospital, Liaocheng, China; ^5^Department of Gastroenterology, Liaocheng People's Hospital, Liaocheng, China; ^6^NMPA Key Laboratory for Research and Evaluation of Narcotic and Psychotropic Drugs, Xuzhou Medical University, Xuzhou, China; ^7^Department of Anesthesiology, The Affiliated Hospital of Xuzhou Medical University, Xuzhou, China

**Keywords:** anesthesia assistance, colorectal cancer, adenoma detection rate, polyp detection rate, retrospective, propensity score matching

## Abstract

**Background:**

Adenoma detection rate (ADR) and polyp detection rate (PDR) are both indicators for colonoscopy quality. Improving ADR or PDR is critical for reducing the incidence and mortality of colorectal cancer (CRC). Although several studies have focused on identifying the factors that may influence ADR or PDR, the evidence remains limited and inconclusive. We conducted a retrospective study to evaluate the effect of anesthesia assistance (AA) on ADR or PDR in patients undergoing colonoscopy screening and identify risk factors affecting ADR or PDR.

**Methods:**

We reviewed electronic medical records of patients who underwent colonoscopy screening between May 2019 and August 2020. Patients were divided into two groups according to whether they received AA: patients in Group A underwent colonoscopy screening with AA, whereas patients in Group O underwent colonoscopy screening without AA. Propensity score matching (PSM) was utilized to account for differences in baseline characteristics. After, ADR and PDR were compared between the two groups. Binary logistic regression was employed to identify risk factors that affected ADR or PDR.

**Results:**

Of 9432 patients who underwent colonoscopy examination during the study period, 7170 were included in the final analyses (Group A = 5756 and Group O = 1414). After PSM, 736 patients remained in each group for analyses. There was no significant difference between groups A and O (*P* > 0.05) in ADR or PDR. Binary logistic regression indicated that the endoscopic device version (Olympus HQ290), equipment image-based technique and number of images were independent risk factors that affected ADR, and the age (50–59 years and 60–69 years), gender (male), high-risk status, endoscopist seniority (senior endoscopist), equipment image-based technique and number of images were all independent risk factors that affected PDR.

**Conclusions:**

We discovered that AA does not affect ADR or PDR. Despite improved patient satisfaction, using AA is unnecessary for improving colonoscopy quality. Endoscopists should consider all these factors as much as possible when performing colonoscopy screening.

## Introduction

Colorectal cancer (CRC) is one of the most malignant cancers worldwide. CRC incidence has been ranked fifth in China, and the new cases and deaths account for 20% of total global cases (1). Most CRCs can be cured early, and the 5-year survival rate can be as high as 90% (2). As a result, improving the early diagnostic yield of CRC is critical for patient survival.

Colonoscopy screening has been regarded as the preferred choice for detecting colorectal lesions (3, 4). It has been demonstrated that CRC incidence could be reduced by 76–90% following clearing colonoscopy (5). High-quality colonoscopy screening is critical to resolving the issue. Adenoma detection rate (ADR) has been extensively recommended as a key colonoscopy quality indicator (6–8). Removal of adenomas and precancerous lesions could reduce CRC incidence and mortality (9–11). Polyp detection rate (PDR) is another indicator of colonoscopy quality. Numerous studies indicate that PDR can serve as a good surrogate for ADR, with an average ADR to PDR ratio of 0.64 to 0.68 (12–15). As a result, investigations on how to improve ADR or PDR are worthwhile.

Certain factors influence the technical performance of colonoscopy screening in clinical practice, of which anesthesia assistance (AA) may be one of the major factors. Although several studies focused on the effect of sedation on ADR or PDR, the findings remained limited and inclusive. Accordingly, we employed a retrospective approach to observe whether AA could increase ADR or PDR in patients undergoing colonoscopy screening. This study aimed to assess the influence of AA on ADR or PDR using propensity score matching (PSM) analyses. Using binary logistic regression analyses, the secondary objective was to identify risk factors affecting ADR or PDR.

## Methods

### Study Population

All electronic records of patients were obtained from the Digestive Endoscopy Center of Liaocheng People's Hospital. Patients were included in the study if they underwent a colonoscopy screening between May 2019 and August 2020. Exclusion criteria included the following: inpatients, emergency patients, patients with bleeding, Boston bowel preparation scale (BBPS) score = 0; patients undergoing endoscopic treatment, patients aged <40 years, patients with intestinal obstruction, and patients undergoing intestinal resection surgery. This study was approved by the Ethics Committee of Liaocheng People's Hospital (Ethics number: 2021098).

### Variables and Outcome Measurements

We obtained the following information from the electronic record system (Medcare Digital Digestive Endoscopy Workstation, Medcare Digital Engineering Co., Ltd, Qingdao, China): age, gender, endoscopist seniority, endoscopic device version, image-enhanced endoscopy, number of endoscopic images, whether the patient is a high-risk population of CRC, biopsy pathological results, recipient of AA or not, BBPS score, and cecum intubation rate (CIR).

Before colonoscopy screening, all patients underwent 12 h of fasting and polyethylene glycol for bowel preparation. BBPS score was used to determine the quality of bowel preparation (16). Each of the three segments of colon (right, including the cecum and ascending colon; transverse, including hepatic and splenic flexures; and left, including the descending colon, sigmoid, and rectum) is given a score from 0 to 3 defined as follows: 0 = unprepared colon segment with mucosa not observed due to inability to pass solid stool; 1 = portion of mucosa of the colon segment observed, but other areas of colon segment, not well-observed due to staining, residual stool, and/or opaque liquid; 2 = minor amount of residual staining, small fragments of stool and/or opaque liquid, but mucosa of colon segment well observed; 3 = entire mucosa of colon segment well-observed with no residual staining, small fragments of stool, or opaque liquid (16).

Anesthesiologists performed AA using propofol or midazolam-fentanyl according to standard AA guidelines set by the institute. The anesthesiologist adjusted the amount of anesthetic medication administered according to the clinical situation and was not collected in this study. All included patients were divided into two groups (A or O) according to whether they received AA. Patients in Group O did not receive any drug-related to anesthesia.

All colonoscopy screenings were performed by 25 endoscopists with a minimum of 500 colonoscopy examinations who had ≥ 1-year experience before initiating this study. Six endoscopists were classified into a junior group, 11 into an intermediate group, and eight into a senior group based on their intensive training time. All endoscopists in the senior group were experts who underwent intensive training for ≥ 5 years.

All colonoscopy screenings were performed using Sonoscape or Olympus with five versions of 550 (EG-550; Sonoscape, Shenzhen, China), 260 (Q260, H260; Olympus, Tokyo, Japan), and 290 (H290, HQ290; Olympus, Tokyo, Japan) series. HQ290 series was classed as high-definition. The procedure details were recorded on an endoscopy database (Medcare Digital Digestive Endoscopy Workstation, Medcare Digital Engineering Co., Ltd, Qingdao, China).

In our study, the term “high-risk population” referred to those with a high risk of CRC. People with a high risk of CRC included those with blood in the stool, melena, anemia, weight loss, CRC warning symptoms, and those aged 50–74 without CRC warning symptoms. Any one of the following is considered a high-risk group: 1) fecal occult blood positive; 2) first-degree relatives have a history of colorectal cancer; 3) past history of intestinal adenoma; 4) history of cancer; 5) changes in bowel habits; 6) those who met any two of the following: chronic diarrhea, chronic constipation, mucus blood in the stool, history of chronic appendicitis or appendectomy, history of chronic cholecystitis or cholecystectomy, long-term mental depression, and alarm signal.

All biopsy pathological results were reviewed, and diagnoses were made by two experienced pathologists. Difficult cases were confirmed after discussions between pathologists in the digestive subspecialty group. We interpreted the findings using the following definitions.

ADR is defined as the percentage of colonoscopies with at least one adenoma, whereas PDR is defined as the percentage of colonoscopies with at least one polyp.

The primary endpoint of the study was to observe the difference in ADR between groups A and O as well as PDR. The secondary endpoint was to identify the factors influencing ADR or PDR.

### Statistical Analyses

The sample size was based on the available data from patients who underwent colonoscopy screening at the Digestive Endoscopy Center of Liaocheng People's Hospital from May 2019 to August 2020. The sample size was not statistically calculated because the parameters necessary to estimate the sample size cannot be determined in advance without references, and as an exploratory study, the number of cases collected is sufficient. As appropriate, the study results are presented as numbers (percentages) for categorical variables and means ± standard deviations (SD) for continuous variables. The normality of data was assessed using the normal quantile-quantile plot. To compare continuous and categorical variables between groups, independent samples *t*-tests and chi-square tests were employed, respectively.

PSM was utilized to reduce the potential confounding effect of each variable and assess differences in baseline characteristics between the two groups. The logistic regression analyses defined the propensity score as the probability of receiving AA. The variables used for matching included age, gender, endoscopist seniority, endoscopic device version, and high-risk status that appear to influence the probability of receiving AA. We matched patients at a ratio of 1:1 using the nearest neighbor method with a caliper of 0.0002 of the logit of the propensity score. In the propensity-matched cohort, paired chi-square and paired rank-sum tests were employed to compare the paired groups.

Binary logistic regression models were performed to identify the risk factors that affected ADR or PDR. All variables were adjusted in the binary logistic regression analyses using the enter method to assess the association between AA and ADR or PDR. To satisfy the linear relationship between age and dependent variable (Logit P), the patients were categorized into the following age groups: 40–49 years, 50–59 years, 60–69 years, 70–79 years, and ≥ 80 years.

The data were analyzed using SPSS software (version 26.0; SPSS Inc., Armonk, NY, United States) and reviewed by a statistician.

## Results

**Figure 1:**
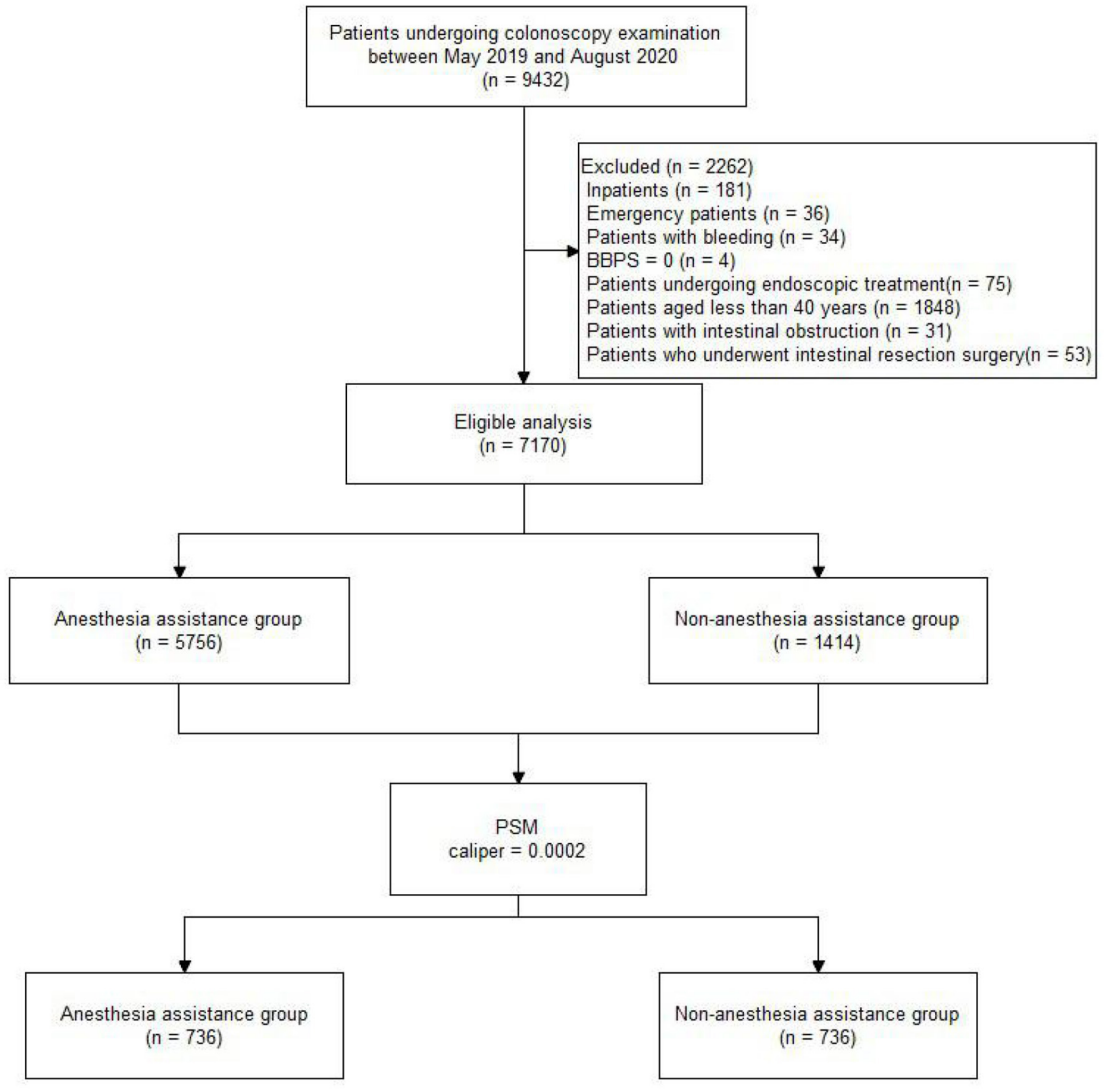
Flow diagram of the study population. BBPS, Boston bowel preparation scale; PSM, Propensity score matching.

Of 9432 patients who underwent colonoscopy screening between May 2019 and August 2020, 7170 were included in the final analyses. The patients who underwent colonoscopy screening with and without AA were allocated to groups A (5756, 80%) and O (1414, 20%), respectively. Figure 1 displays the distribution of patients, and Table 1 shows the characteristics of the total study cohort.

**Table 1:** Patient characteristics of the total study cohort.

**Characteristic**	**Group A (*****n*** **=** **5756)**		**Group O (*****n*** **=** **1414)**		***P-*values**
	***n***	**Proportion (%)**	***n***	**Proportion (%)**	
Age, years					<0.001
40–49 years	1576	27.4%	466	33.0%	
50–59 years	2222	38.6%	544	38.5%	
60–69 years	1398	24.3%	311	22.0%	
70–79 years	515	8.9%	89	6.3%	
≥ 80 years	45	0.8%	4	0.3%	
Gender					<0.001
Male	2731	52.6%	805	56.9%	
Female	3025	47.4%	609	43.1%	
High risk population of CRC	823	14.3%	206	14.6%	0.800
Endoscopist seniority					<0.001
Junior endoscopist	496	8.6%	560	39.6%	
Intermediate endoscopist	3725	64.7%	653	46.2%	
Senior endoscopist	1535	26.7%	201	14.2%	
Endoscopic device version					<0.001
Sonoscape EC-550	225	3.9%	901	63.7%	
Olympus Q260	2557	44.4%	240	17.0%	
Olympus H260	1935	33.6%	179	12.7%	
Olympus H290	297	5.2%	15	1.1%	
Olympus HQ290	742	12.9%	79	5.6%	
Image-enhanced endoscopy					<0.001
WLE	4039	70.2%	1165	82.4%	
Equipment image-based techniques	1685	29.3%	246	17.4%	
Dyed-based technique	32	0.6%	3	0.2%	
Number of endoscopic images	77.10 ± 29.674		79.15 ± 32.663		<0.001
BBPS score	7.02 ± 1.326		6.78 ± 1.190		<0.001
CIR	5673	98.6%	1380	97.6%	0.014
Adenoma	661	11.5%	129	9.1%	0.010
Polyp	2473	43.0%	552	39.0%	0.007
SSA/P	154	2.7%	28	2.0%	0.157
LST	15	0.3%	3	0.2%	1.000
Advanced cancer	149	2.6%	36	2.5%	1.000
IBD	83	1.4%	33	2.3%	0.025

*Data are presented as the median (range) or number (percentage)*.

*CRC, Colorectal cancer; WLE, white light endoscopy; BBPS, Boston bowel preparation scale; CIR, Cecum intubation rate*.

*SSA/P, Sessile serrated adenomas/polyps;LST, Laterally spreading tumor; IBD, Inflammatory bowel disease*.

Following PSM with a caliper of 0.0002, 736 patients remained in each group, which was well-matched except for the endoscopic device version (*P* < 0.05). Table 2 indicates no significant difference in ADR or PDR (*P* > 0.05) between groups A and O in the propensity-matched cohort. Meanwhile, no significant difference was observed in detection rates of sessile serrated adenomas/polyps (SSA/P), laterally spreading tumor (LST), and advanced cancer (*P* > 0.05, Table 2). There was a significant difference in inflammatory bowel disease (IBD) (*P* < 0.05, Table 2).

**Table 2:** Patient characteristics of the propensity-matched cohort.

**Characteristic**	**Group A (*****n*** **=** **736)**		**Group O (*****n*** **=** **736)**		***P-*values**
	***n***	**Proportion (%)**	***n***	**Proportion (%)**	
Age, years					0.948
40–49 years	245	33.3%	239	32.5%	
50–59 years	262	35.6%	274	37.2%	
60–69 years	181	24.6%	174	23.6%	
70–79 years	45	6.1%	47	6.4%	
≥ 80 years	3	0.4%	2	0.3%	
Gender					0.251
Male	375	51.0%	353	48.0%	
Female	361	49.0%	383	52.0%	
High risk population of CRC	103	14.0%	101	13.7%	0.880
Endoscopist seniority					0.183
Junior endoscopist	171	23.2%	186	25.3%	
Intermediate endoscopist	411	55.8%	423	57.5%	
Senior endoscopist	154	20.9%	127	17.3%	
Endoscopic device version					<0.001
Sonoscape EC-550	224	30.4%	224	30.4%	
Olympus Q260	240	32.6%	240	32.6%	
Olympus H260	179	24.3%	178	24.2%	
Olympus H290	43	5.8%	15	2.0%	
Olympus HQ290	50	6.8%	79	10.7%	
Image-enhanced endoscopy					<0.001
WLE	473	64.3%	599	81.4%	
Equipment image-based techniques	258	35.1%	135	18.3%	
Dyed-based technique	5	0.7%	2	0.3%	
Number of endoscopic images	76.52 ± 31.716		71.93 ± 28.379		0.003
BBPS score	6.94 ± 1.286		6.87 ± 1.182		0.264
CIR	722	98.1%	724	98.4%	0.692
Adenoma	80	10.9%	69	9.4%	0.342
Polyp	301	40.9%	266	36.1%	0.061
SSA/P	14	1.9%	13	1.8%	0.846
LST	1	0.1%	3	0.4%	0.317
Advanced cancer	19	2.6%	20	2.7%	0.871
IBD	9	1.2%	20	2.7%	0.039

*Data are presented as the median (range) or number (percentage)*.

*PSM, Propensity score matching; CRC, Colorectal cancer; WLE, white light endoscopy; BBPS, Boston bowel preparation scale; CIR, Cecum intubation rate; SSA/P, Sessile serrated adenomas/polyps; LST, Laterally spreading tumor; IBD, Inflammatory bowel disease*.

In the propensity-matched cohort, univariate analyses were employed to screen for variables that may affect ADR or PDR, and the results revealed that age, gender, high-risk status, equipment image-based technique, the number of images, endoscopic device version, and endoscopist seniority were all significant variables (*P* < 0.05). Subsequently, a binary logistic regression model was constructed to evaluate the association between AA and ADR or PDR. After controlling for confounding variables, the results indicated that AA does not affect ADR (*P* = 0.906) or PDR (*P* = 0.770).

For ADR, the results revealed that the endoscopic device version (Olympus HQ290), equipment image-based technique, and number of images were independent risk factors (Table 3). ADR was 2.166 times higher in patients who underwent colonoscopy with Olympus HQ290 than Sonoscape EG-550 (95% CI: 1.058–4.433, *P* = 0.034). Compared with colonoscopy with white light endoscopy (WLE), ADR was 2.326 times higher in patients who underwent colonoscopy with equipment image-based technique (95% CI: 1.565–3.459, *P* < 0.001). For every additional endoscopic image, ADR increased by 1.009-fold (95% CI: 1.003-1.015, *P* = 0.002). In addition, it has been demonstrated that ADR was unaffected by AA, age, gender, high-risk status, endoscopist seniority, endoscopic device version (Olympus Q260, H260, H290), dyed-based technique, and BBPS score (Table 3).

**Table 3:** Logistic regression analysis for ADR in the propensity-matched cohort.

	**Detection rate, %**	**Unadjusted**			**Adjusted**		
		**OR**	**95% CI**	***P*–value**	**OR**	**95% CI**	***P*–values**
Colonoscopy without AA	9.4%	Reference			Reference		
Colonoscopy with AA	10.9%	1.179	0.839–1.656	0.342	0.978	0.680–1.408	0.906
Age							
40–49 years	8.7%	Reference			Reference		
50–59 years	9.7%	1.131	0.738–1.732	0.573	1.157	0.744–1.799	0.518
60–69 years	13.0%	1.567	1.006–2.439	0.047	1.354	0.849–2.159	0.204
70–79 years	9.8%	1.141	0.535–2.433	0.733	1.005	0.454–2.224	0.990
≥ 80 years	0	/	/	/	/	/	/
Gender							
Female	7.3%	Reference			Reference		
Male	12.9%	1.887	1.327–2.684	<0.001	1.451	1.000–2.106	0.050
High risk population							
None	8.9%	Reference			Reference		
High risk population of CRC	17.6%	2.190	1.456–3.295	<0.001	1.548	0.985–2.431	0.058
Endoscopist seniority							
Junior endoscopist	9.8%	Reference			Reference		
Intermediate endoscopist	9.0%	0.909	0.596–1.386	0.658	0.631	0.373–1.068	0.086
Senior endoscopist	13.9%	1.483	0.912–2.410	0.112	1.190	0.654–2.168	0.569
Endoscopic device version							
Sonoscape EG−550	7.8%	Reference			Reference		
Olympus Q260	10.0%	1.311	0.831–2.069	0.244	1.491	0.838–2.651	0.174
Olympus H260	11.8%	1.573	0.981–2.522	0.060	1.733	0.965–3.113	0.066
Olympus H290	10.3%	1.362	0.547–3.392	0.508	1.257	0.453–3.484	0.661
Olympus HQ290	14.0%	1.914	1.044–3.507	0.036	2.166	1.058–4.433	0.034
Image–enhanced endoscopy							
WLE	6.8%	Reference			Reference		
Equipment image–based technique	18.8%	3.175	2.244–4.492	<0.001	2.326	1.565–3.459	<0.001
Dyed–based technique	28.6%	5.474	1.044–28.702	0.044	4.715	0.843–26.375	0.077
Number of images	/	1.011	1.006–1.016	<0.001	1.009	1.003–1.015	0.002
BBPS score	/	1.014	0.884–1.163	0.841	0.926	0.801–1.071	0.303

*Data are presented as the number (percentage)*.

*ADR, Adenoma detection rate; AA, Anesthesia assistance; OR, odd ratio; CI, confidence interval; CRC, Colorectal cancer; WLE, white light endoscopy; BBPS, Boston bowel preparation scale*.

For PDR, the results revealed that age (50–59 years and 60–69 years), gender (male), high-risk status, endoscopist seniority (senior endoscopist), equipment image-based technique, and number of images were all independent risk factors (Table 4). Compared with patients of 40–49 years, PDR was 1.510 times higher in those of 50–59 years (95% CI: 1.134–2.011, *P* = 0.005) and 1.879 times higher in those of 60–69 years (95% CI: 1.370–2.576, *P* < 0.001). Compared with female patients, PDR was 1.622 times higher in male patients (95% CI: 1.278–2.058, *P* < 0.001). Compared with patients without a high risk of CRC, PDR was 1.857 times higher in those with a high risk of CRC (95% CI: 1.310-2.632, *P* = 0.001). Compared with colonoscopy performed by a junior endoscopist, PDR was 1.547 times higher in patients who underwent colonoscopy performed by a senior endoscopist (95% CI: 1.039–2.304, *P* = 0.032). Compared with colonoscopy with WLE, PDR was 3.210 times higher in patients who underwent colonoscopy with equipment image-based technique (95% CI: 2.421–4.255, *P* < 0.001). For every additional endoscopic image, PDR increased by 1.017-fold (95% CI: 1.012–1.021, *P* = 0.001). In addition, PDR was unaffected by AA, age (70-79 years and ≥ 80 years), endoscopist seniority (intermediate endoscopist), endoscopic device version, dyed-based technique, and BBPS score (Table 4).

**Table 4:** Logistic regression analysis for PDR in the propensity–matched cohort.

	**Detection rate, %**	**Unadjusted**			**Adjusted**		
		**OR**	**95% CI**	***P*–value**	**OR**	**95% CI**	***P*–values**
Colonoscopy without AA	36.1%	Reference			Reference		
Colonoscopy with AA	40.9%	1.223	0.991–1.509	0.061	0.965	0.759–1.227	0.770
Age							
40–49 years	30.6%	Reference			Reference		
50–59 years	38.6%	1.428	1.101–1.853	0.007	1.510	1.134–2.011	0.005
60–69 years	48.2%	2.110	1.588–2.803	<0.001	1.879	1.370–2.576	<0.001
70–79 years	43.5%	1.746	1.108–2.753	0.016	1.463	0.876–2.442	0.146
≥ 80 years	20.0%	0.568	0.063–5.121	0.614	0.411	0.043–3.906	0.439
Sex							
Female	29.9%	Reference			Reference		
Male	46.9%	2.067	1.668–2.561	<0.001	1.622	1.278–2.058	<0.001
High risk population							
None	35.3%	Reference			Reference		
High risk population of CRC	58.3%	2.562	1.896–3.463	<0.001	1.857	1.310–2.632	0.001
Endoscopist seniority							
Junior endoscopist	38.7%	Reference			Reference		
Intermediate endoscopist	36.2%	0.901	0.698–1.163	0.423	0.835	0.604–1.153	0.272
Senior endoscopist	45.2%	1.309	0.953–1.797	0.096	1.547	1.039–2.304	0.032
Endoscopic device version							
Sonoscape EG−550	38.2%	Reference			Reference		
Olympus Q260	37.9%	0.989	0.759–1.290	0.937	0.983	0.700–1.380	0.920
Olympus H260	38.7%	1.021	0.767–1.359	0.888	1.047	0.730–1.502	0.803
Olympus H290	41.4%	1.143	0.656–1.994	0.637	0.775	0.403–1.493	0.446
Olympus HQ290	40.3%	1.094	0.733–1.632	0.660	1.156	0.714–1.871	0.555
Image–enhanced endoscopy							
WLE	29.2%	Reference			Reference		
Equipment image–based technique	63.4%	4.193	4.193–5.351	<0.001	3.210	2.421–4.255	<0.001
Dyed–based technique	71.4%	6.062	6.062–31.412	0.032	5.424	0.900–32.675	0.065
Number of images	/	1.019	1.015–1.023	<0.001	1.017	1.012–1.021	0.001
BBPS score	/	0.961	0.883–1.046	0.357	0.874	0.793–0.963	0.077

*Data are presented as the number (percentage)*.

*PDR: Polyp detection rate; AA, Anesthesia assistance; OR, odd ratio; CI, confidence interval; CRC, Colorectal cancer; WLE, white light endoscopy; BBPS, Boston bowel preparation scale*.

## Discussion

In this study, no significant difference exists in ADR or PDR between groups A and O. Binary logistic regression analyses revealed that the endoscopic device version (Olympus HQ290), equipment image-based technique, and number of images were independent risk factors that affected ADR, and the age (50–59 years and 60–69 years), gender (male), high-risk status, endoscopist seniority (senior endoscopist), equipment image-based technique, and number of images were all independent risk factors that affected PDR.

Colonoscopy screening is extremely important because it is linked to a substantially reduced incidence of CRC (17, 18). Numerous studies have proved the remarkable efficacy of colonoscopy in preventing cancer (19–25). The protective effect of CRC is achieved by comprehensively detecting and subsequently resecting precancerous lesions via colonoscopy. Although colonoscopy screening has been demonstrated to reduce CRC-related mortality (26), interval cancer (IC), which occurred between consecutive inspections, still hampered the benefit of colonoscopy screening. It has been shown that IC represents up to 9% of all patients diagnosed with CRC (27–30). A study discovered that ADR was inversely associated with IC risks (31). Each 1.0% increase in ADR was linked to a 3.0% decrease in CRC risk (31). In addition, PDR, with a much simpler calculation than ADR, is another valuable quality indicator of colonoscopy. According to Sastre Lozano VM, IC prevalence was inversely and strongly associated with PDR of endoscopists (32). Therefore, ADR and PDR have been widely advocated as important quality indicators for colonoscopy screening.

Due to their fear of imaginary pain, numerous patients selected colonoscopy screening with AA. Once the patients are sedated, it is easy to fill more air and allow the endoscopist to thoroughly inspect the mucosa. However, sedation will bring more adverse problems, such as falling tongue, aspiration, and even hypoxia. Although many studies focus on the impact of sedation on ADR or PDR, the results remain controversial. A retrospective study of 48,838 procedures revealed that sedation is linked to increased CIR, but ADR and PDR remain unchanged (33). Christina Bannert has revealed that sedation does not increase ADR or PDR (34). Another randomized controlled trial demonstrated no difference in PDR when colonoscopy was performed under deep or moderate sedation (35). Compared with colonoscopist-administrated sedation, using AA did not affect ADR of trainees (36). These findings are consistent with our finding that AA does not affect ADR or PDR during colonoscopy screening. However, some studies reached the opposite conclusion. Fatima Khan revealed that colonoscopy with sedation, as opposed to no sedation, was significantly linked to higher ADR (37). Qiongmei Zhang indicated that sedation was an independent factor associated with higher ADR (38). A study revealed that midazolam/fentanyl sedation administered by colonoscopists might increase PDR during colonoscopy (39). These findings are contrary to our results. On the one hand, because these studies were all retrospective, the results may be biased. In our study, we adopt PSM to reduce the influence of confounding factors, hence minimizing bias. On the other hand, previous studies did not consider the impact of endoscopic device version, image-enhanced endoscopy, or a high-risk status, but we have considered all these factors in our study. The reason that AA could not affect ADR or PDR during colonoscopy screening is possible due to the following: 1) compared with esogastroduodenoscopy, the pain associated with colonoscopy screening is tolerant for patients; 2) sedation makes the patient's body position changes impossible, which may affect the detection rate of lesions; and 3) small doses of opioids used in AA could not decrease the bowel movement which may affect the detection rate of lesions during a colonoscopy screening.

There are still many other factors affecting ADR. Binary logistic regression analyses revealed that the endoscopic device version (Olympus HQ290) is an independent risk factor affecting ADR, which was 2.166 times higher than Sonoscape EG-550, consistent with previous research results. Ashley Bond observed that colonoscopy screening with Olympus HQ290 could improve ADR within the moderate-risk population (40). Narrow-band imaging (NBI) is one of the equipment image-based techniques. Our study demonstrated that equipment image-based technique is an independent risk factor affecting ADR. Compared with colonoscopy with WLE, ADR was 2.326 times higher in patients who underwent colonoscopy with equipment image-based technique. A meta-analysis revealed that NBI has a higher ADR than WLE when bowel preparation is optimal (41). Irina Ioana Vi?ovan found that ADR in NBI group was significantly higher than non-NBI group (35.3% vs. 20%) (42), consistent with our study. On the contrary, Tatsunori Minamide revealed that second-generation NBI could not surpass WLI in terms of ADR but improved the detection of easily overlooked flat and depressed lesions (43). The reason may be that patients enrolled in this study are over 20 years old, which is different from our study, which included patients over 40 years. The incidence of lesions is higher in patients over 40 years. A study indicated that longer withdrawal times were linked to higher ADR (44). In our study, we could not obtain data regarding colonoscopy examination time. The endoscopic images were taken by an endoscopist during the withdrawal procedure so that the number of images could reflect the withdrawal time to some extent. As a result, we used the number of endoscopic images as a proxy for examination time in our study and found a 1.009-fold increase in ADR with every additional image. However, the overall ADR in our study is 11.5%, lower than that recommended by the current guidelines (45, 46). The reason may be that the withdrawal times of some endoscopists were too short to find adenoma. In addition, Margaret J. Zhou found that increasing age was independently associated with ADR, and female sex was inversely correlated with ADR (47, 48). However, it is inconsistent with our study that ADR was unaffected by age and gender. The reason may be attributed to different sample sizes and inclusion criteria. Although ADR has been established as a quality target in average-risk individuals, high-risk status was unrelated to ADR (49). It is consistent with our study that ADR was unaffected by high-risk status. We also found that ADR was unaffected by endoscopist seniority. It is consistent with the result of a prior study that ADR was not significantly associated with any endoscopist characteristic (50). Our study also found that ADR was unaffected by dyed-based technique, consistent with prior studies that ADR was not improved by the dyed-based technique (51, 52). ADR has been associated with bowel preparation but is not proportional to BBPS. ADR is the highest in good bowel preparation rather than excellent preparation (53, 54). It is consistent with our study that ADR was unaffected by BBPS. The reason may be that residual stool may help the endoscopist pay attention to the mucosa and thus identify lesions.

For PDR, binary logistic regression analyses revealed that age (50–59 years and 60–69 years) is an independent risk factor. Compared with patients of 40–49 years, PDR was 1.510 times higher in those of 50–59 years and 1.879 times higher in those of 60–69 years. A cross-sectional study found that PDR is significantly higher in most patients after the age of 50, without providing detailed statistics on PDR for each age group (55). This study also found that the percentage of male patients with polyps was significantly higher than that of female patients (55), consistent with our study that PDR was 1.622 times higher in male patients. Our study stated that PDR was 1.857 times higher in patients with a high-risk of CRC than those without a high-risk of CRC. The reason may be that the lifestyle risk factor for polyps and CRC partially overlaps (56). Da Kyoung Jung demonstrated that PDR increased when colonoscopy was performed by experienced endoscopists (57). It is consistent with our study that PDR was 1.547 times higher in patients who underwent colonoscopy performed by a senior endoscopist. Our study revealed that PDR was 3.210 times higher in patients who underwent colonoscopy with equipment image-based technique, consistent with a prior study that increased PDR for NBI colonoscopy (42). As in the previous discussion of ADR, we use the number of endoscopic images instead of the withdrawal time. For every additional endoscopic image, PDR increased by 1.017-fold. It is consistent with the prior study, in which longer withdrawal times were associated with higher PDR (44). In addition, our study demonstrated that PDR was unaffected by the endoscopic device version. It is inconsistent with a prior study that using high-definition equipment was the most important factor associated with a higher PDR (58). The inconsistency may be attributed to different inclusion criteria and study populations in our study. In addition, PDR has been demonstrated to be unaffected by BBPS score, consistent with a prior study in which PDR is the highest in good bowel preparation rather than excellent preparation (53). As in the previous discussion of ADR, the reason may be that residual stool may help the endoscopist pay attention to the mucosa and thus identify lesions.

Several limitations should be considered when interpreting the results of this study. First, this work was a retrospective study. Although we employed PSM to control for confounding variables, many real cases were missed. This will result in increasing internal validity while reducing external validity. Second, we used the number of endoscopic images as an alternative since colonoscopy examination time was unavailable and found a 1.009-fold increase in ADR and 1.017-fold increase in PDR, respectively, with every additional image. However, this could be the result rather than the cause of increased detection. There were more adenomas or polyps, and many images were taken to document the findings. This may affect our findings. Third, AA included sedation with propofol or midazolam-fentanyl, which were not classified separately. We will conduct further research to address this issue in the future.

## Conclusions

In summary, we demonstrate that ADR or PDR, important indicators for colonoscopy screening quality, are unaffected by AA. The results also revealed that endoscopic device version (Olympus HQ290), equipment image-based technique, and number of images were independent risk factors that affected ADR. All independent risk factors that affected PDR included the following: age (50–59 years and 60–69 years), gender (male), high-risk status, endoscopist seniority (senior endoscopist), equipment image-based technique, and number of images. Despite improved patient satisfaction, using AA is unnecessary for improving colonoscopy quality. Endoscopists should consider all these factors as much as possible when performing colonoscopy screening.

## Data Availability Statement

The raw data supporting the conclusions of this article will be made available by the authors, without undue reservation.

## Ethics Statement

The studies involving human participants were reviewed and approved by Ethics Committee of Liaocheng People's Hospital. Written informed consent for participation was not required for this study in accordance with the national legislation and the institutional requirements.

## Author Contributions

ML and CX collected data and wrote the manuscript. XZ assisted in collecting literature and participated in discussions. ML reviewed the statistical analyses. ZZ and JC designed the study. ZZ examined and verified the study. All authors read and approved the final manuscript.

## Conflict of Interest

The authors declare that the research was conducted in the absence of any commercial or financial relationships that could be construed as a potential conflict of interest.

## Publisher's Note

All claims expressed in this article are solely those of the authors and do not necessarily represent those of their affiliated organizations, or those of the publisher, the editors and the reviewers. Any product that may be evaluated in this article, or claim that may be made by its manufacturer, is not guaranteed or endorsed by the publisher.

## Abbreviations

ADR: Adenoma detection rate
PDR: Polyp detection rate
CRC: Colorectal cancer
AA: Anesthesia assistance
PSM: Propensity score matching
BBPS: Boston bowel preparation scale
CIR: Cecum intubation rate
SSA/P: Sessile serrated adenomas/polyps
LST: Laterally spreading tumor
IBD: Inflammatory bowel disease
IC: Interval cancer
WLE: White light endoscopy
NBI: Narrow-band imaging.
